# Unique and efficient adsorbents for highly selective and reverse adsorption and separation of dyes *via* the introduction of SO_3_H functional groups into a metal–organic framework†

**DOI:** 10.1039/c9ra10840h

**Published:** 2020-03-04

**Authors:** Mahdie Saghian, Saeed Dehghanpour, Massoomeh Sharbatdaran

**Affiliations:** A Department of Chemistry, Alzahra University P. O. Box 1993891176 Tehran Iran Dehghanpours@alzahra.ac.ir +982188041344; Nuclear Sciences and Technology Research Institute Karaj Iran

## Abstract

In this study, an unsaturated Cu-based MOF, HKUST (Cu_3_(BTC)_2_), was fabricated and modified with sulfonate groups in two steps, leading to the construction of a novel sulfo-functionalized MOF. The prepared framework was utilized in the adsorption and separation of various organic dyes (MB, Er, FS, and MV). The adsorption process represented intriguing features due to the introduction of the SO_3_H functional groups into the framework. Such an attractive feature has rarely been depicted in previous works. In addition to the substantially increased adsorption capacity of the modified framework compared with that of pristine MOF, a reverse and selective phenomenon was perceived in the cases of FS and MV. The sulfo-functionalized MOF could adsorb MV with high adsorption capacity but barely adsorbed FS, and the opposite condition was observed for pristine MOF. In addition, the prepared framework showed high selectivity in a mixed solution of dyes. On the other hand, the modified framework had no role in the first step of the adsorption and separation process and showed the same behavior as pristine MOF. Furthermore, the sulfonate functional groups could not be directly incorporated into HKUST. The experimental data followed the pseudo-second-order kinetics and the Langmuir isotherm model. Thermodynamic studies demonstrated an exothermic spontaneous mechanism for the dye adsorption process. The prepared adsorbents were capable of being recycled for four sequential cycles. Hereupon, this study presents a notably efficacious approach for the reverse performance of frameworks for the dye adsorption and separation process.

## Abstract 2

A novel sulfo-functionalized MOF was utilized as an efficient adsorbent for a reversal in the removal and selective separation of dyes from contaminated water.

## Graphical abstract

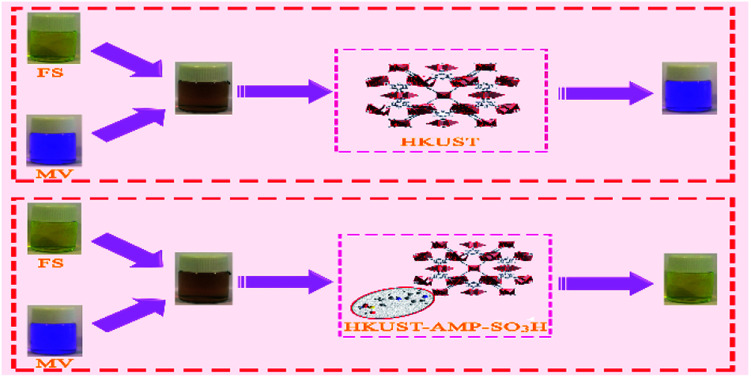

## Introduction

Nowadays, dyes are applied in various industries, such as leather, paper, pharmaceutical, textiles, cosmetics and food.^1–3^ Despite the great applications of these compounds in the industry, they have some disadvantages: they are highly stable, non-degradable, toxic and carcinogenic.^4^ Even a small amount of these compounds in wastewater can lead to serious hazards to the environment and human health. Accordingly, finding an appropriate treatment method to eliminate these toxic compounds from contaminated water is significantly imperative. There are numerous methods for the removal of dyes from wastewater, including biodegradation,^5,6^ oxidation,^7^ adsorption,^8^ and filtration.^9,10^ Among these widespread methods, adsorption is the most commonly implemented technique for the removal of dyes from effluent water due to its advantages, such as the simplicity of the operation process, easy sequestration, regeneration, and low cost.

Various kinds of adsorbents, such as activated carbon,^11,12^ montmorillonite,^13^ chitosan,^14,15^ clay minerals,^16^ zeolites,^17,18^ and metal–organic frameworks^19–21^ (MOFs), have been applied to eliminate dye molecules from aqueous media. Among the aforementioned adsorbents, MOFs have gained significant attention in recent years owing to their unique characteristics.^22^ MOFs are a new class of porous crystalline structures, which constitute metal clusters and multifunctional organic ligands. They have attracted much attention among other porous structures due to their intriguing and unique properties, including high porosity and surface area, diverse accessible sites, high thermal stability, and tunable pore size.^23–25^ These features make MOFs good candidates for various applications, such as adsorption,^26,27^ catalysis,^28,29^ drug delivery,^30,31^ separation,^32^ and sensing.^33^ Although various methods have been applied to enhance the dye adsorption capacity of MOFs, the effect of functional groups on the reverse and selective adsorption and separation of dyes has rarely been investigated.^34,35^ Post-synthetic modification (PSM) is one of the impressive methods to increase the performance of the framework. Overall, PSM methods for the functionalization of MOFs include PSM on the organic linker, PSM on the unsaturated metal centers (UMC) and encapsulation within the cavities of the structure (Scheme S1†). Various ligands can adhere to the unsaturated metal centers and improve the properties of these structures.

In this regard, the adsorption and separation properties of some dyes, including Methylene Blue (MB), Methyl Violet (MV), Fluorescein sodium (FS), and Erythrosine (Er), over MOFs have been investigated in this work. For this purpose, HKUST was synthesized and modified with SO_3_H functional groups. By introducing SO_3_H groups into the framework, intriguing features were introduced in the structure. The functionalized MOF triggered not only a substantial increment in the adsorption capacity of the above-mentioned dyes but also a reversible and selective state for the adsorption towards MV and FS. The details of the adsorption isotherms and kinetic characteristics are discussed.

## Experimental methods

### Materials and instrumentation

Materials and instrumentation are explained in the ESI.†

### Synthesis of adsorbents

#### Synthesis of Cu_3_(BTC)_2_ (HKUST – Hong Kong University of Science and Technology)

The preparation of HKUST was carried out by a previously reported method.^36^ In a typical procedure, copper(ii) nitrate trihydrate (875 mg, 3.6 mmol) was dissolved in 12 mL of deionized water, and trimesic acid (420 mg, 2 mmol) was dissolved in 12 mL of ethanol. The solutions were mixed and transferred to a 50 mL Teflon-lined steel autoclave and heated at 120 °C for 12 h. The resulting blue crystals were separated and washed with a mixture of deionized water and ethanol. To activate the as-synthesized compound, the solid was heated at 150 °C for 24 h under vacuum, and the color changed from turquoise blue to violet-blue.

#### Synthesis of HKUST-AMP

Activated HKUST (400 mg) was suspended in 15 mL toluene. 4-Aminopyridine (100 mg; 1.06 mmol) dissolved in 20 mL dichloromethane was added to the HKUST solution. The resulting mixture was refluxed for 16 h. The solid obtained was filtered and washed with dichloromethane to remove the excess ligand and dried at 100 °C for 3 h.

#### Synthesis of HKUST-AMP-SO_3_H

530 mg of HKUST-AMP was added to a solution containing 1,3-propanesultone (340 mg, 2.78 mmol) in chloroform (30 mL) and stirred at room temperature for 24 h followed by refluxing for 3 h. The resulting precipitate was filtered and soxhleted for 2 days in chloroform and dried at 120 °C for 24 h.

#### Dye adsorption experiments

**Scheme 1:**
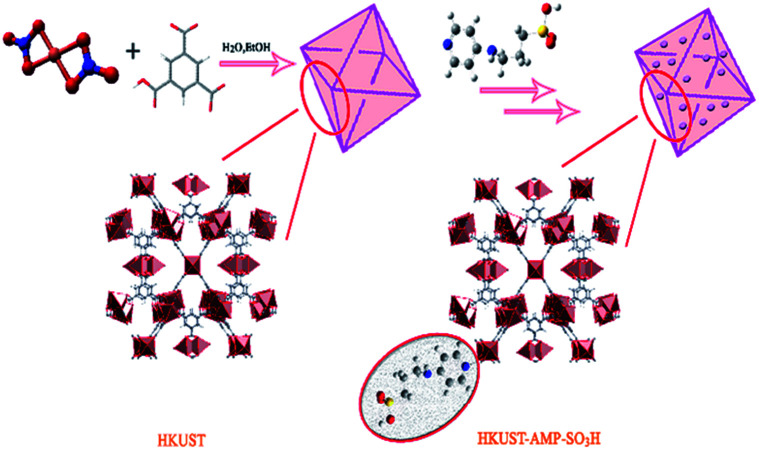
Synthesis of HKUST-AMP-SO_3_H.

The removal of dyes, including MB, MV, FS, and Er, from aqueous solutions was performed by using a batch technique in the presence of the prepared HKUST and HKUST-AMP-SO_3_H frameworks (Scheme 1). Typically, 10 mg of the freshly prepared adsorbent was added to 20 mL of a dye-containing solution with diverse initial concentrations (20–200 mg L^−1^), and the mixture was stirred at room temperature. After the completion of the dye adsorption process, the adsorbent was separated by centrifugation and the concentration of the residual dye in the solution was monitored by means of a UV-Vis spectrophotometer. In addition, the temperature, pH and adsorption time were optimized. The adsorbed amount of the dyes at the equilibrium (*q*_e_) was computed by the following equation:1
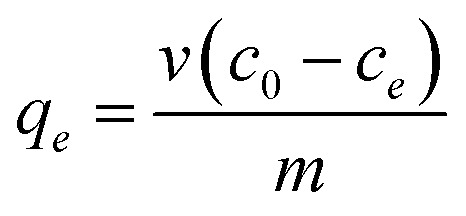


#### Dye separation experiments

To perform the separation process, 10 mg of the prepared adsorbent was added to 20 mL of a mixed aqueous solution of FS and MV with different initial concentrations, and the mixture was stirred at room temperature for 30 min. The residual dye in the solution was monitored by a UV-Vis spectrophotometer.

The competitive adsorption of MV over FS and that for FS over MV was calculated by the adsorption selectivity constants (*α*_MV/FS_ and *α*_FS/MV_), as given by eqn (2) and (3), where *Q*_FS_ and *Q*_MV_ are the adsorption capacities for FS and MV, respectively, and CFS and CMV are the initial concentrations of FS and MV in the two-component solutions, respectively.2
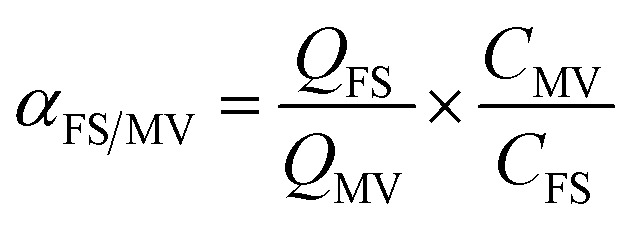
3
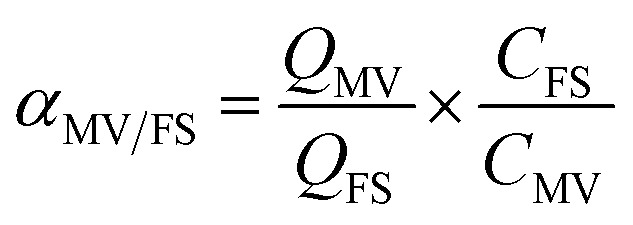


## Results and discussion

### Characterization of adsorbents

The FT-IR spectra of the as-synthesized compounds are presented in Fig. S1.† In the FT-IR spectrum of HKUST (Fig. S1a†), the characteristic bands, which appeared at 1378 and 1642 cm^−1^, were assigned to the symmetric and asymmetric stretching vibrations of the carboxylic acid functional group, respectively.^31^ Moreover, the bands at 728 and 762 cm^−1^ could be ascribed to the out-of-plane vibration of the C–H groups in the phenyl rings.^36^ The FT-IR spectrum of HKUST-AMP is shown in Fig. S1b.† The band related to the stretching vibration of the C=N groups within the pyridine ring overlapped with the asymmetric COO stretching vibrations owing to the high intensity of the relevant band. In contrast, the band at 3364 cm^−1^ was attributed to the N–H stretching vibration of 4-aminopyridine, which affirmed the presence of NH_2_ groups in the framework.^17^ The FT-IR spectrum of HKUST-AMP-SO_3_H is shown in Fig. S1c.† As shown in this figure, the peaks manifested at 1039 and 1200 cm^−1^ could be assigned to the stretching vibrations of the SO_3_^−^ and S=O bonds, respectively.^34^ These relevant bands indicated the successful modification of the framework with the SO_3_H functional group.

The XRD patterns of the synthesized compounds can be observed in Fig. S2.† According to the main diffraction peaks, it could be seen that the XRD patterns of stimulated (Fig. S2a†) and as-synthesized HKUST (Fig. S2b†) were thoroughly identical and in agreement with previous reports, confirming the purity and crystallinity of the framework.^36^ Furthermore, the XRD pattern of HKUST-AMP-SO_3_H (Fig. S2c†) indicated that the modification had caused no changes in the pattern, which confirmed the maintenance of topology and structural stability throughout the modification process.

To determine the thermal and structural stability of the compounds, thermogravimetric analysis (TGA-DSC) was conducted, and the results are illustrated in Fig. S3.† Fig. S3a and b† represent the HKUST and HKUST-AMP-SO_3_H frameworks, respectively. The TGA curves revealed a two-step weight loss for each framework. As observed in Fig. S3a,† the first weight loss of only 6% up to 125 °C was related to the removal of solvent molecules from the framework, which was in agreement with the endothermic peak in the DSC curve. The other weight loss of about 52% was due to the thermal decomposition of MOF. This weight loss occurred at 335 °C and was accompanied by exothermic peaks in the DSC curve. For the modified framework (Fig. S3b†), a weight loss of 11% was observed as a result of the loss of solvent molecules from the framework. As an endorsement of the above, an endothermic peak could be observed. The degradation and collapse of MOF occurred at 300 °C, along with an exothermic peak with 60% weight loss. The residual weights of 42% and 29% could be attributed to the formation of CuO.

To confirm the modification of the framework with SO_3_H groups, the amount of sulfur was measured using a carbon–sulfur analyzer. Based on the results, the amount of sulfur in the framework was 0.28%. Therefore, according to the molecular weight of sulfur, the amount of SO_3_H groups loaded in the framework was 1.1%.

**Fig. 1:**
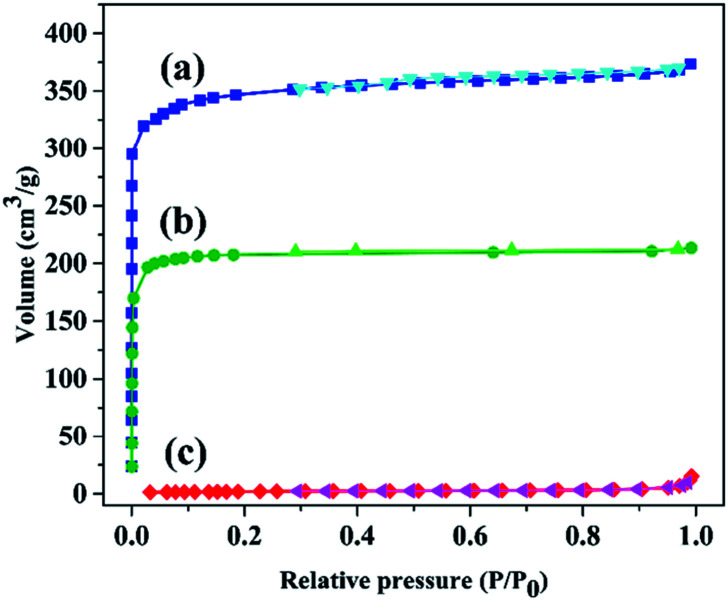
N_2_ adsorption–desorption isotherms of (a) HKUST and HKUST-AMP-SO_3_H (b) before and (c) after dye adsorption.

Moreover, to affirm the successful modification of the framework, N_2_ adsorption–desorption analysis at low temperatures was accomplished. This analysis determines the pore size distribution, surface area, pore volume and the porosity of a compound. The N_2_ adsorption–desorption isotherms for the as-synthesized and modified HKUST and the textural parameters are shown in Fig. 1 and Table 1, respectively. The N_2_ adsorption isotherms of both pristine and modified HKUST exhibited type I/IV isotherm (Fig. 1). As a result, Table 1 shows a substantial decrease in the surface area from 1363 to 896 m^2^ g^−1^ is owing to the modification of the framework. Furthermore, after modification, the pore volume and pore diameter reduced from 0.58 to 0.33 cm^3^ g^−1^ and 1.69 to 1.47 nm, respectively. The aforementioned results clearly confirmed the presence of the SO_3_H functional groups and the successful modification of the framework. Moreover, after dye adsorption, a remarkable decrease in the surface area from 896 to 7.55 m^2^ g^−1^ and pore volume from 0.33 to 0.01 cm^3^ g^−1^, as well as pore diameter from 1.47 to 0.05 nm could be observed due to the presence of dye molecules within the structure. Furthermore, the N_2_ adsorption isotherm displayed a type II isotherm (Fig. 1c). The reduction in the textural parameters and the change in the isotherm type confirmed the adsorption of the desired dye on the structure.

**Table 1:** Textural parameters of HKUST and HKUST-AMP-SO_3_H

Entry	Sample	*S*_BET_ (m^2^ g^−1^)	Pore volume (cm^3^ g^−1^)	Pore diameter (nm)
1	HKUST	1363	0.5773	1.6942
2	HKUST-AMP-SO_3_H	895.59	0.33	1.4739
3	HKUST-AMP-SO_3_H after MB adsorption	7.5583	0.019	1.045

**Fig. 2:**
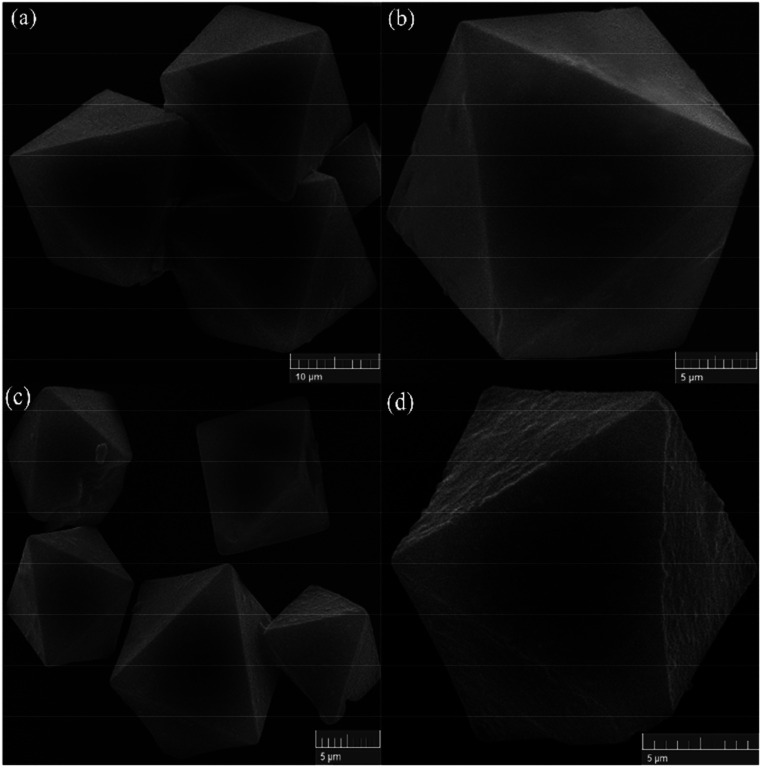
SEM images of (a and b) HKUST and (c and d) HKUST-AMP-SO_3_H.

Scanning electron micrographs of HKUST and HKUST-AMP-SO_3_H are presented in Fig. 2a–d. The as-synthesized HKUST showed an octahedral morphology with an average diameter of around 6 micrometers (Fig. 2a), and the modification of the compound caused no changes in the morphology of the structure (Fig. 2c).

### Dye adsorption study of the synthesized compounds

To investigate the adsorption efficiency of the synthesized compounds, the adsorption of dyes, including MB, MV, FS, and Er, was studied using HKUST and HKUST-AMP-SO_3_H as the adsorbents. Moreover, the effects of reaction time, dye concentration, pH, and temperature on the adsorption performance were also evaluated.

### Dye removal ability of the synthesized compounds

Dye removal from contaminated water was calculated by the following equation:4
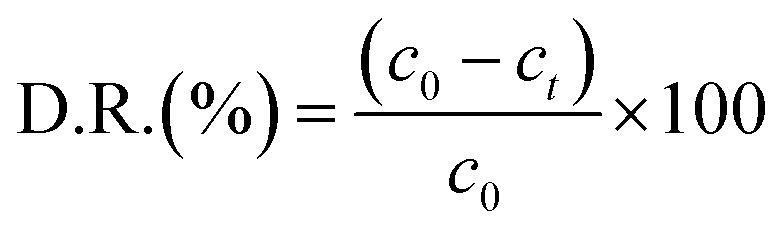


The adsorption capacity and the average amounts of dyes removed at an optimized time are shown in Table 2. For MB and Er, high dye removal percentages and adsorption capacities were observed owing to the introduction of SO_3_H groups into the framework compared with those of the pristine framework, whereas the reverse status could be seen for MV and FS. In this regard, HKUST could adsorb FS, but hardly adsorbed MV. On the contrary, HKUST-AMP-SO_3_H could adsorb MV but rarely adsorbed FS. The reasons for these phenomena are discussed subsequently.

**Table 2:** The dye removal ability of the synthesized frameworks

Adsorbent	Dye removal%, (adsorption capacity)			
	MB	MV	FS	Er
HKUST	64 (238.09)	3 (14)	81 (243.90)	46 (200)
HKUST-AMP-SO_3_H	97 (833.33)	85 (714.28)	9 (33)	72 (833.33)

### Effect of contact time on dye adsorption

**Fig. 3:**
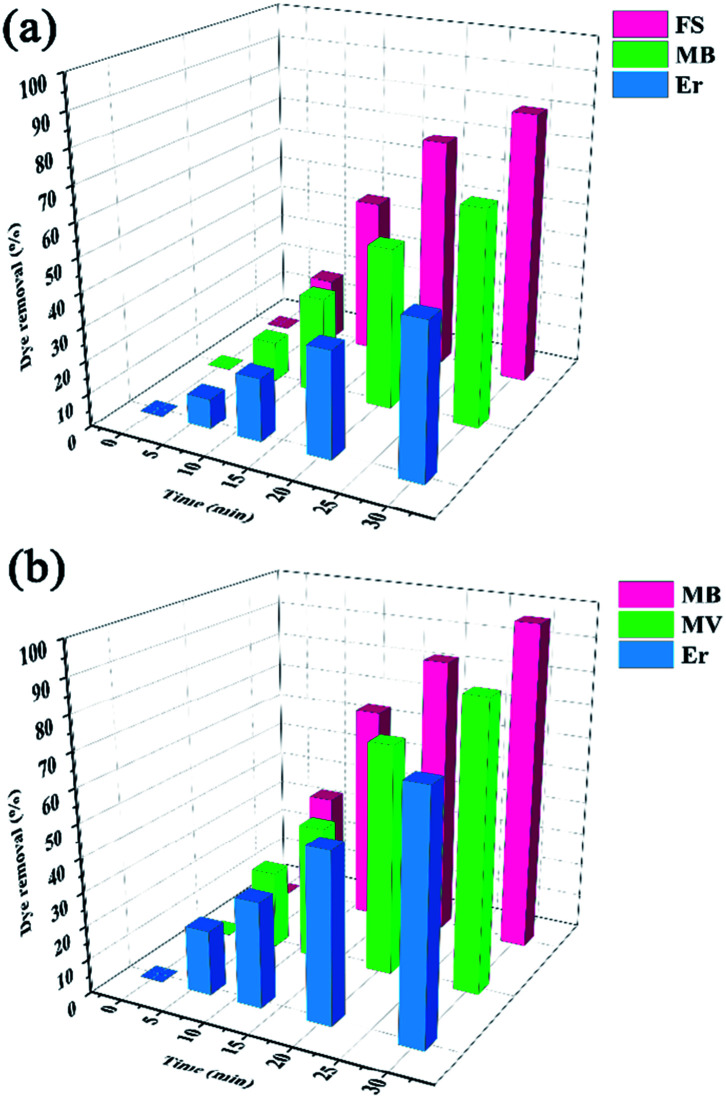
Effect of contact time on dye removal over (a) HKUST and (b) HKUST-AMP-SO_3_H.

The removal of MB, MV, FS, and Er was evaluated by HKUST and HKUST-AMP-SO_3_H at room temperature. As seen in Fig. 3, an increase in the reaction time increased dye adsorption. However, after a certain time, the amount of adsorption did not increase and remained constant (Fig. S4†). This time was referred to as the equilibrium time for the reactions. The adsorbent could not adsorb the desired dye anymore beyond this time due to the saturation of active sites. Based on the results in Fig. 3, the equilibrium time for HKUST (Fig. 3a) and HKUST-AMP-SO_3_H (Fig. 3b) was obtained as 30 min. The fast adsorption of various dyes indicated the presence of vacant or suitable sites for adsorption.

### Dye concentration effect on the adsorption of dye

Table S1† shows the amount of dye removed using HKUST and HKUST-AMP-SO_3_H as adsorbents with different initial dye concentrations. It is obvious from the results that dye removal reduced on increasing the initial dye concentration, which might be due to the occupation of active sites of the adsorbent. This was true for both frameworks.

### Influence of pH on dye adsorption

It is acknowledged that the solution pH, which is a vital factor during the adsorption process, can influence the ionization degree and the structure of the adsorbate molecules, as well as the active site charge of the adsorbent.^15^ Nevertheless, in this study, the pH of the solution was investigated in the range of 3–11. The solution pH was adjusted using 0.1 M HCl or 0.1 M NaOH, and the results are presented in Fig. S5.† It could be observed that the adsorption capacity and subsequently, dye removal were enhanced on increasing the solution pH from 3 to 7 and decreased upon increasing the solution pH further. Therefore, the favorable pH value for the adsorption process using HKUST and HKUST-AMP-SO_3_H was 7.

### Effect of temperature on dye adsorption

To elucidate the effect of temperature, adsorption experiments were performed at diverse temperatures in the presence of HKUST and HKUST-AMP-SO_3_H as the adsorbents, and the results are shown in Table S2.† The results indicated that dye removal reduced upon increasing the temperature from 298 to 323 K. Thus, the room temperature was the appropriate temperature for the adsorption process. Adsorption isotherms with respect to the temperature are discussed later.

### Effect of adsorbent dosage

Table S3† shows the amounts of dye removed with different adsorbent dosages. It was clear from the results that dye removal increased upon increasing the adsorbent dose from 3 to 10 mg and remained constant with a further increase up to 20 mg. Therefore, the best results were obtained with 10 mg of adsorbents.

### Adsorption kinetics

Adsorption kinetics is one of the important factors to understand the adsorption process better.^14^ Adsorption kinetics is mainly used to examine the effect of time on the removal of adsorbates, as well as the diffusion mechanism. Two adsorption kinetic models were used for this purpose, including pseudo-first-order and pseudo-second-order equations, which are explained in the ESI.†

The adsorption kinetics of HKUST and HKUST-AMP-SO_3_H were evaluated through the adsorption of the desired dyes at different contact times, and the results are presented in Fig. S4, S6 and S7.† The investigation of adsorption kinetics was performed at various concentrations. Moreover, the correlation coefficient values (*R*_2_) obtained from the fitting line (Fig. S6 and S7†) and the kinetic adsorption parameters are listed in Tables S4 and S5.† As observed in Fig. S6, S7, Tables S4 and S5,† the results fitted the pseudo-second-order kinetics model well, showing higher *R*_2_ values for the pseudo-second-order model compared with those of the pseudo-first-order model. Accordingly, the pseudo-second-order model, the most appropriate model to express the adsorption behavior of the dyes over the desired adsorbent, represented chemisorption as the mechanism of the adsorption process.

### Adsorption isotherms

Adsorption isotherms are used to illustrate the equilibrium state of the adsorbates between the solid and liquid phases and understand the interactions between the adsorbent surface and adsorbate molecules. There are several isotherm models for dye adsorption, of which Langmuir and Freundlich's models have been investigated and explained in the ESI.†

In order to evaluate the adsorption isotherms, the adsorption of desired dyes was carried out over the adsorbents at three different temperatures (298, 308, and 318 K) in the initial concentration range of dyes from 20 to 200 mg L^−1^. According to the results presented in Fig. S8, S9, Tables S6 and S7,† the correlation coefficient *R*_2_ values for the adsorption of MB, FS, and Er over HKUST fitted the Langmuir isotherm model. The same results were obtained for HKUST-AMP-SO_3_H. Langmuir's theory demonstrates that the adsorption process occurs uniformly and in a monolayer form.

### Adsorption thermodynamics

The study of the thermodynamic parameters of the adsorption mechanisms is necessary in order to evaluate the desirability of the adsorption process. The values of thermodynamic parameters are explained in the ESI.†

To study the thermodynamic parameters, adsorption experiments were performed at three different temperatures (298, 308, and 318 K) in the presence of the adsorbents, and the results are presented in Tables S8 and S9.† The negative Δ*G* values represented the spontaneity of the dye adsorption process. The absolute value of Δ*G* was in accordance with the adsorption capacity and enhanced with increasing adsorption capacity. The high absolute value of Δ*G* confirmed that the modification of the framework was efficacious in improving the performance.

### Dye separation process

**Fig. 4:**
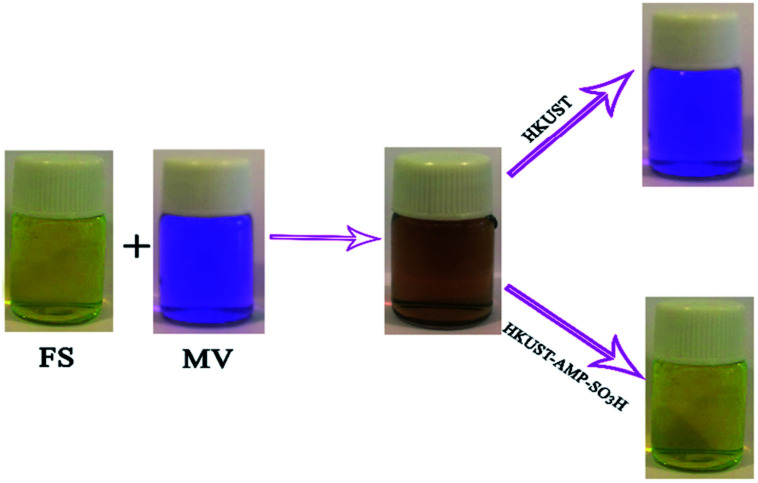
The reverse performance of HKUST and HKUST-AMP-SO_3_H for FS–MV mixture.

**Fig. 5:**
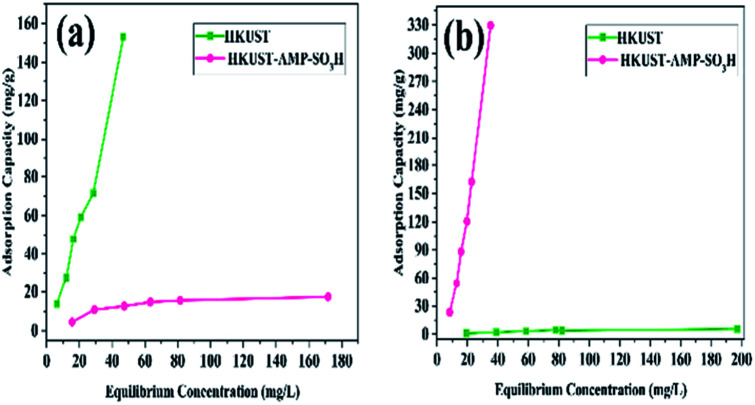
Adsorption capacity of HKUST and HKUST-AMP-SO_3_H for (a) FS and (b) MV in two-component solution.

HKUST and HKUST-AMP-SO_3_H were employed with a mixed solution of FS and MV. According to Fig. 4, the brown mixture turned pink and fluorescent green in the presence of HKUST and HKUST-AMP-SO_3_H, respectively. Therefore, the incorporation of SO_3_H functional groups into the framework caused a reversal in the dye separation process. This feature is very different from previously reported works on dye separation. The adsorbance of MV and FS by HKUST and HKUST-AMP-SO_3_H from the mixed solution are represented in Fig. S10,† which confirmed the aforementioned feature. Furthermore, the adsorption capability of HKUST and HKUST-AMP-SO_3_H to adsorb MV and FS at the equilibrium concentration are shown in Fig. 5, from which the reversal process in dye adsorption can be observed.

**Fig. 6:**
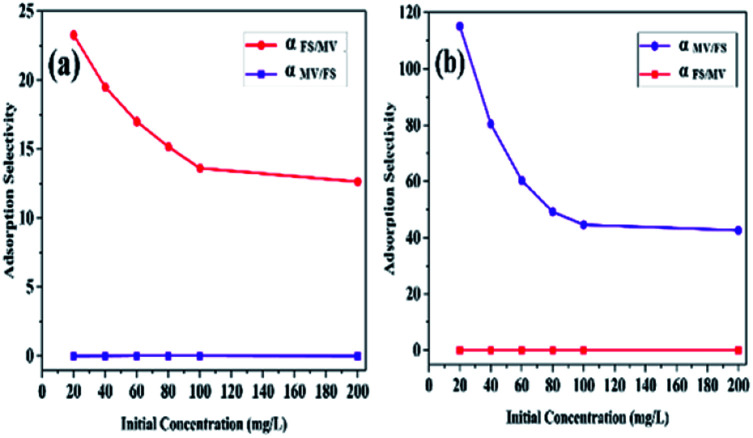
The adsorption selectivity of (a) HKUST and (b) HKUST-AMP-SO_3_H with different initial dye concentrations.

Furthermore, the adsorption selectivity constants obtained from the competitive experiments in two-component solutions with different initial concentrations are shown in Fig. 6. As shown in Fig. 6, HKUST preferentially showed selective adsorption of FS over MV in all the two-component solutions. On the other hand, with the introduction of SO_3_H groups into the framework, reverse selectivity was observed, and the framework preferentially exhibited selective adsorption of MV over FS in all the two-component solutions. Moreover, the selectivity constant *α*_FS/MV_ of HKUST decreased with an increase in the initial concentrations, while under the same condition, the selectivity constant *α*_MV/FS_ of HKUST remained at a very low value, and for HKUST-AMP-SO_3_H, opposite selectivity could be observed.

### The plausible mechanism of the adsorption process

Various mechanisms have been presented below to determine the process of adsorption and removal of dyes in different reports.^19,34,37^ As mentioned above, in our work, two states were observed in the dye adsorption process after the modification of the framework.

(i) A remarkable increase in the adsorption capacity and dye removal was observed owing to the modification of the framework with SO_3_H groups in the case of MB and Er (Table 3, entries 1 and 4).

**Table 3:** The ability to adsorb diverse adsorbates using HKUST and HKUST-AMP-SO_3_H as adsorbent

Entry	Dye	Structure	Adsorbent	
			HKUST	HKUST-AMP-SO_3_H
1	Methylene Blue	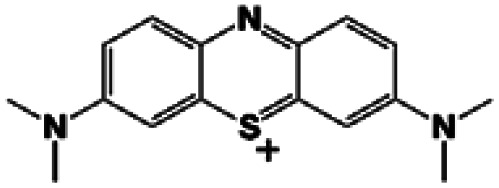	✓	✓
2	Methyl Violet	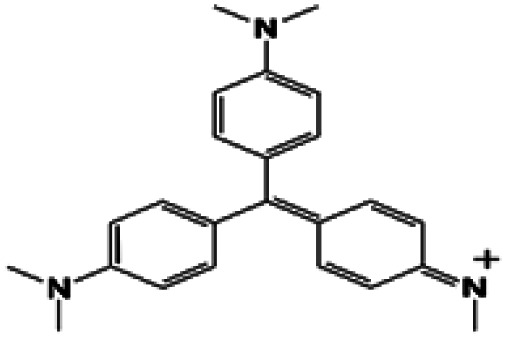	✗	✓
3	Fluorescein Sodium	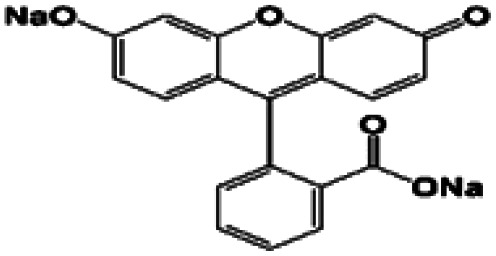	✓	✗
4	Erythrosine	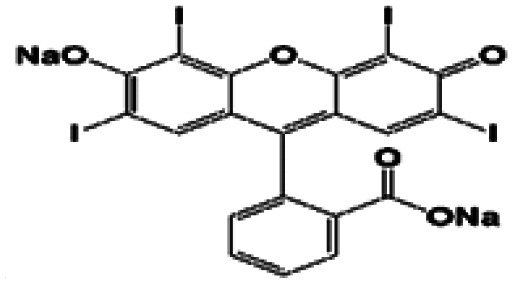	✓	✓

(ii) A marvelous feature of reverse adsorption was observed *via* the introduction of SO_3_H groups into the framework in the case of MV and FS (Fig. 4 and Table 3, entries 2 and 3).

The aforementioned features can be explained as follows.

Overall, the adsorption process can be carried out by π–π interactions, electrostatic interactions, or hydrogen bonding.^38^

**Scheme 2:**
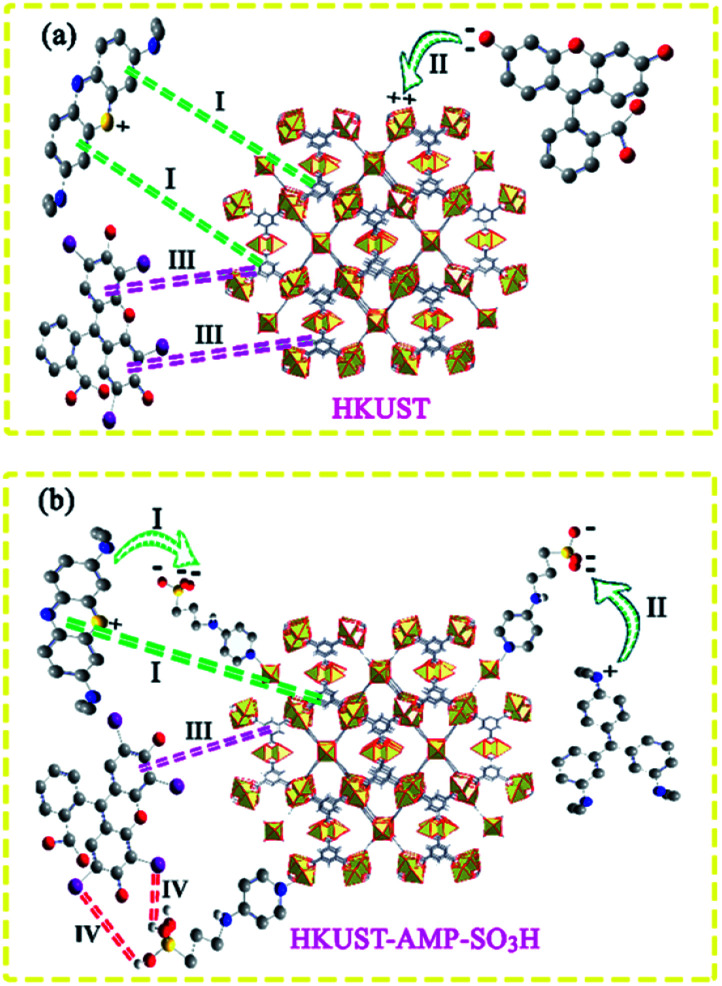
The plausible mechanism for adsorption process of (a) HKUST (b) HKUST-AMP-SO_3_H.

In the case of MB, it could be seen that both HKUST and HKUST-AMP-SO_3_H adsorbed the desired dye. HKUST and HKUST-AMP-SO_3_H have the zeta potentials of −4.03 and −17.8 mV, respectively. According to the zeta potential, when HKUST is applied as the adsorbent, both π–π and electrostatic interactions drive the adsorption process (Scheme 2a, pathway I). However, upon the application of HKUST-AMP-SO_3_H as the adsorbent, in addition to π–π interactions, more electrostatic interactions occur, which is an effective factor in the adsorption process. After the modification of the framework with sulfopropyl groups, negatively charged sulfonate and positively charged ammonium groups exist in the zwitterionic form of the MOF. This negative charge increases the electrostatic interactions between the framework and the cationic dye molecules (Scheme 2b, pathway I), and thus, increases the adsorption capacity compared with that of the pristine MOF. On the other hand, the surface charge becomes more negative upon modification, which is in accordance with the zeta potential of HKUST-AMP-SO_3_H. Therefore, the zeta potential measurement confirmed that electrostatic interactions play an important role in the adsorption process and increase the adsorption capacity.

In entries 2 and 3 of Table 3, inverse situations are observed for MV and FS. For MV, HKUST-AMP-SO_3_H is the appropriate adsorbent since it is capable of adsorbing the MV dye molecules with a high adsorption capacity. On the contrary, HKUST can hardly adsorb MV (Fig. 4). This phenomenon can be attributed to the positive charge on the surface of the dye molecules, which facilitates adsorption on HKUST-AMP-SO_3_H (Scheme 2b, pathway II). Therefore, the higher negative charge on the surface of HKUST-AMP-SO_3_H improves the adsorption process, which is in accordance with the zeta potential of the modified framework. On the other hand, the Fluorescein dye showed the reverse condition. It could be adsorbed by HKUST but only scarcely by HKUST-AMP-SO_3_H. The more negative zeta potential of HKUST-AMP-SO_3_H causes the reduction in the adsorption of FS.

When Er is used as the dye, both frameworks showed the ability to adsorb the dye, and a considerable increase in the adsorption capacity could be observed after the modification of the framework with the SO_3_H functional groups. In this case, in addition to π–π interactions in both frameworks, hydrogen bonding due to the presence of iodide atoms on the surface of the dye molecules played a significant role in increasing the dye adsorption on HKUST-AMP-SO_3_H (Scheme 2b, pathway IV). FS and Er molecules have the same structures, and the only difference between these two molecules is the presence of iodine atoms on the Er structure, which caused Er to be adsorbed easily on HKUST-AMP-SO_3_H, while FS was rarely adsorbed. Therefore, the introduction of the SO_3_H functional groups into the framework causes the reversal of the performance of MOF toward dye adsorption.

### Reusability of the synthesized adsorbent and its activity

One of the most challenging issues during the adsorption process is the reusability of adsorbents, which makes them good candidates for commercial applications. For this purpose, the reusability of the prepared adsorbents was investigated through consecutive adsorption experiments under the same conditions. After each cycle, the relevant adsorbent was separated, dispersed in a 0.1 M HCl/methanol (1 : 9, v/v) solution to remove the trapped and adsorbed dyes, washed with ethanol, dried at 120 °C, and then used for the subsequent experiment. The results reported in Fig. S11† show that the prepared adsorbent can be reused for four times without any considerable loss in the adsorption efficiency of the pristine MOF. The FT-IR spectra and PXRD patterns (Fig. S12 and S13†) confirmed these findings. Therefore, the prepared adsorbents presented good regeneration ability.

To investigate the activity of the prepared adsorbent (HKUST-AMP-SO_3_H), the synthesized compound and previously reported MOFs were compared in terms of their adsorption capacity, and the results are summarized in Table 4. It could be observed that our adsorbent was remarkably different and had a high adsorption capacity compared to others.

**Table 4:** The comparison of the adsorptive activities of some previously reported adsorbents

Entry	Adsorbent	Adsorbate	Adsorption capacity (mg g^−1^)	Ref.
1	UIO-66-0.75(COOH)_2_	MB	291	37
2	MIL-100(Cr)	MB	645.3	39
3	NH_2_-MIL-125(Ti)	MB	400	40
4	[Zn(phenDIB)(AOBTC)0.5]	MB	139.4	41
5	Fe_3_O_4_@AMCA-MIL53(Al)	MB	325.62	42
6	ZnMOF	MB	30.14	19
7	GO/PAM	MB	292.84	43
8	MOF-235	MB	252	44
9	POM@MIL-101	MB	371	45
10	HKUST-AMP-SO_3_H	MB	833.33	Present work

Furthermore, the adsorption and separation behavior of the modified framework in the first step was also investigated. The results showed that the adsorption behavior of the modified framework in the first step was quite similar to that of the pristine MOF, except that the amount of adsorption was lower due to the blockage of unsaturated metal centers by the amine functional groups. In addition, 1,3-propanesultone was added directly to HKUST to identify the coordination capability of the sulfonate groups. The amount of sulfur was measured using a carbon–sulfur analyser, and no sulfur was detected in the framework, which confirmed that sulfonate functional groups did not directly incorporate into the HKUST structure.

## Conclusion

In summary, a metal–organic framework with an unsaturated metal center, denoted as HKUST, was constructed and successfully modified by the introduction of SO_3_H groups into the framework. The desired framework was applied as an effective adsorbent in the removal and separation of dye molecules from contaminated water. The modification of the framework with SO_3_H groups caused intriguing features in the composition. In addition to the substantial enhancement in the adsorption capacity compared with that of the pristine MOF, a reverse trend was observed in dye adsorption and separation due to the introduction of SO_3_H groups into the framework. This is a completely different feature and has rarely been observed in previously reported adsorbents. Notably, selective adsorption was seen toward cationic dyes owing to the presence of the anionic SO_3_^−^ groups in the framework. The experimental data agreed well with the pseudo-second-order kinetics and the Langmuir isotherm model. The thermodynamic parameters indicated a spontaneous process for dye adsorption. Moreover, the synthesized adsorbents could be recycled for four sequential cycles. Ultimately, these unique characteristics of the desired adsorbent make it a good candidate for applications in dye adsorption and separation processes.

## Conflicts of interest

There are no conflicts to declare.

## Supplementary Material

RA-010-C9RA10840H-s001
