# Supplementary material for: Unique and efficient adsorbents for highly selective and reverse adsorption and separation of dyes via the introduction of SO3H functional groups into a metal–organic framework
Source: RSC Adv. 2020 Mar 4;10(16):9369–77. doi: 10.1039/c9ra10840h (PMC9050141; doi:10.1039/c9ra10840h)
Supplement: RA-010-C9RA10840H-s001 [file RA-010-C9RA10840H-s001.pdf]

Electronic Supplementary Material (ESI) for RSC Advances.
This journal is © The Royal Society of Chemistry 2020

Supporting information

Materials and instrumentation

All reagents were purchased from Sigma-Aldrich and Merck chemical companies and used without further purification. FT-IR spectra were recorded on a Bruker Tensor 27 FT-IR spectrophotometer using KBr pellets over the range of 4000–400 cm^{-1}. The X-ray powder diffraction (XRD) data were recorded on a Siefert XRD 3003 PTS diffractometer, using Cu $K_{\alpha 1}$ radiation (k = 1.5406 Å). UV-Vis spectra were obtained with a shimadzu UV-260 spectrophotometer. Scanning electron microscopy (SEM) images were obtained on a Philips XL-30ESEM equipped with an X-ray energy dispersive detector. Thermogravimetric analysis (TGA) was performed using a Mettler Toledo TGA/DSC instrument with heating rate of 10 °C/min in an air atmosphere. Nitrogen sorption isotherms were recorded on a Belsorp Mini–II instrument at 77K. The amount of sulfur was determined using an ELTRA carbon-sulfur analyzer.

Adsorption kinetics

The pseudo-first-order is based on the following equation:

$$log\left(q_e - q_t\right) = log\, q_e - \frac{k_1}{2.303}t \qquad (1)$$

Where q_e and q_t (mg/g) are the amounts of dye adsorbed at equilibrium and contact time (t), respectively and K_1 (min^{-1}) represents the rate constant of pseudo-first-order kinetics.

The pseudo-second-order model is generally represented as follows:

$$\frac{t}{q_t} = \frac{1}{k_2 q_e^2} + \frac{1}{q_e}t \qquad (2)$$

Where K_2 ($g.mg^{-1}\ min^{-1}$) denotes the adsorption constant of pseudo-second-order kinetics.

Adsorption isotherms

The Langmuir isotherm is expressed by the following equation:

$$\frac{c_e}{Q_e} = \frac{1}{Q_{max}} + \frac{1}{k_L Q_{max}} \qquad (3)$$

Where C_e is the equilibrium concentration of the dye in the solution ($mg.g^{-1}$), Q_e represents the amount of the adsorbed dye at equilibrium (mg/g), Q_{max} denotes the maximum adsorption capacity (mg/g), and K_L is the Langmuir constant (L/g) which is related to the adsorption binding energy. The Freundlich model is represented as:

$$ln\, Q_e \text{ - } Lnk_F = \frac{1}{n} ln\, C_e \qquad (4)$$

Where K_F and n are Freundlich adsorption constants which are attributed to the heterogeneous surface of the adsorbent and the desirability of the adsorption, respectively. If n is greater than 1, the adsorption is favorable and in the case of n<1, the adsorption is unfavorable.

Adsorption thermodynamics

The values of thermodynamic parameters including the changes in enthalpy (ΔH°; kJ/mol), entropy (ΔS°; kJ/mol), and Gibbs free energy (ΔG°; kJ/mol) were computed by the following equations:

$$LnK_0 = \frac{\Delta S^{\circ}}{R} - \frac{\Delta H^{\circ}}{RT} \qquad (5)$$

$$-RT\, LnK_0 = \Delta H^{\circ} - T\, \Delta S^{\circ} \qquad (6)$$

$$\Delta G^{\circ} = \Delta H^{\circ} - T\, \Delta S^{\circ} \qquad (7)$$

Where K_0 is the thermodynamic equilibrium constant, T is the solution temperature (K), and R is the universal gas constant (8.314 J/mol.K) and the plots of LnK_0 vs. 1/T are shown in Fig. S14.

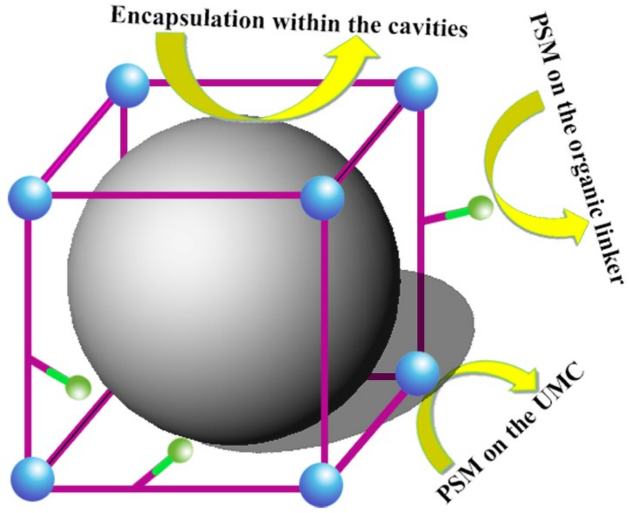

Scheme S1. Various situation for post synthetic modification of MOFs.

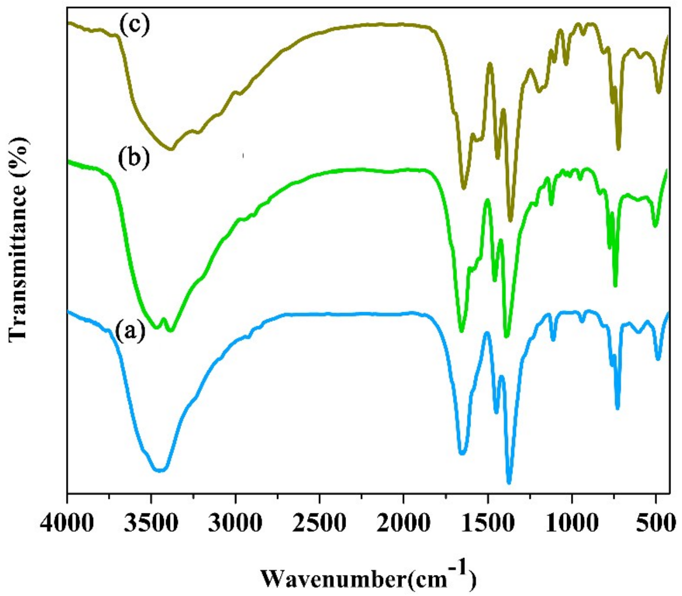

Fig. S1. FT-IR spectra of (a) HKUST (b) HKUST-AMP (c) HKUST-AMP-SO_3H.

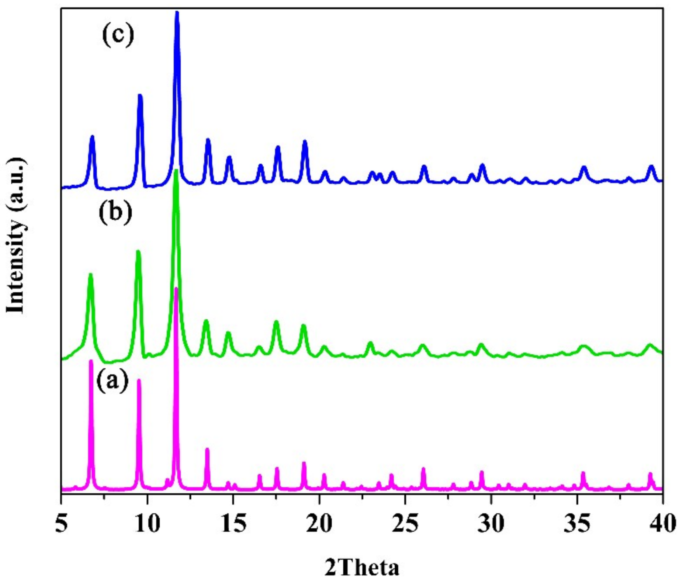

Fig. S2. XRD patterns of HKUST (a) simulated (b) as-synthesized, and (c) HKUST-AMP-SO_3H.

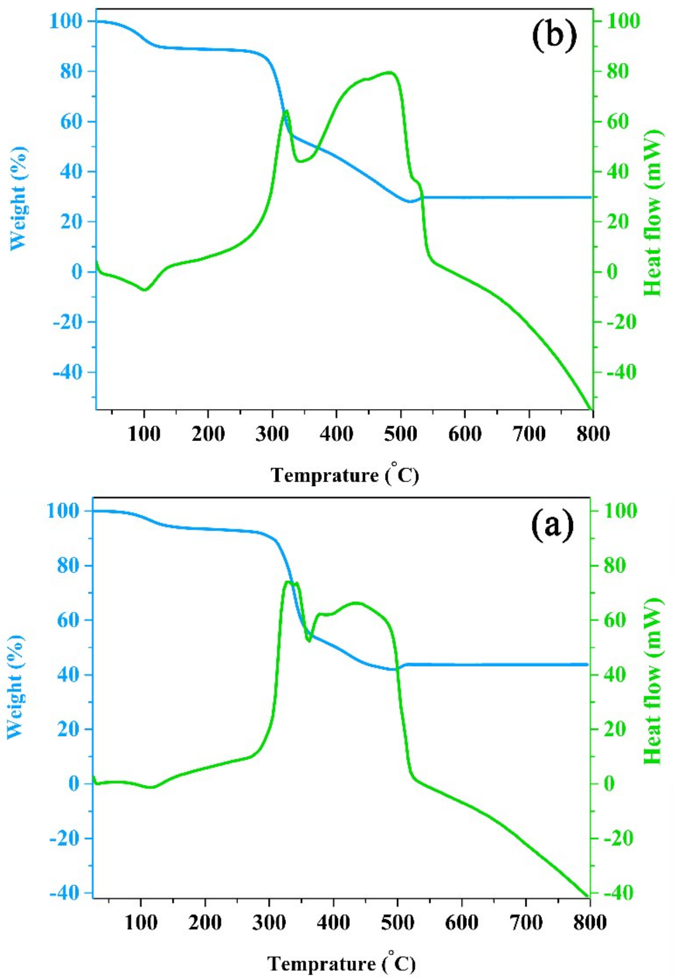

Fig. S3. TGA-DSC curves of (a) HKUST (b) HKUST-AMP-SO_3H.

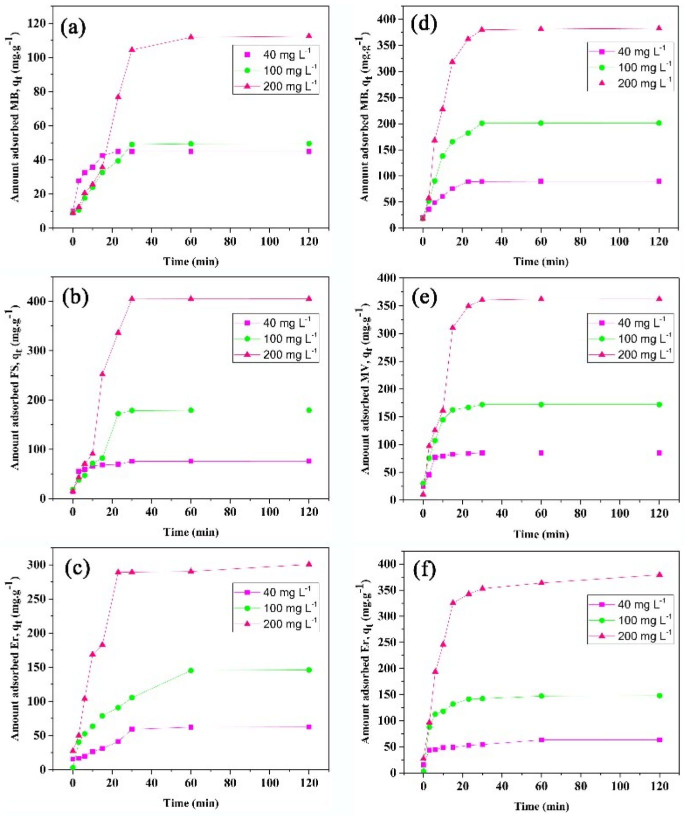

Fig. S4. Adsorption Capacity over HKUST for a) MB, b) FS, and c) Er. Adsorption capacity over HKUST-AMP-SO_3H for d) MB, e) MV, and f) Er.

Table S1. The dye removal percentage of dyes using HKUST and HKUST-AMP-SO_3H in different concentration of dye molecules.

C_0(mg L^{-1})	Dye removal (%)					
	HKUST			HKUST-AMP-SO_3H		
	MB	FS	Er	MB	MV	Er
20	64	81	46	97	85	72
40	60	80	42	95	84	71
60	55	79	39	90	83	70
80	51	76	36	87	82	68
100	49	75	31	86	80	65
200	45	73	20	86	78	60

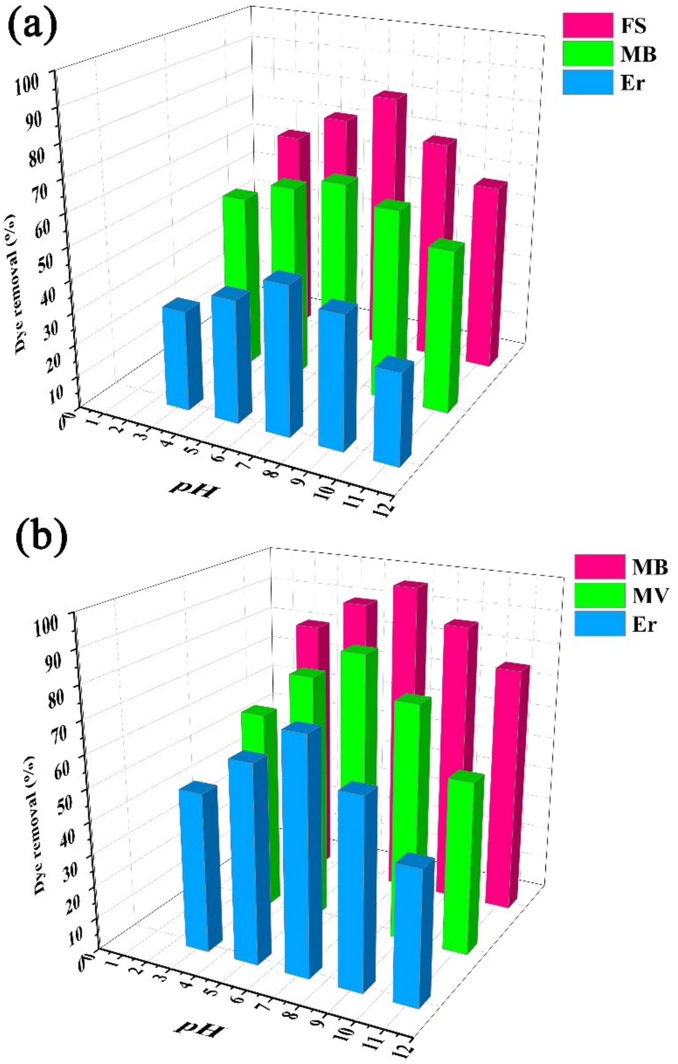

Fig. S5. Effect of pH on dye removal using (a) HKUST (b) HKUST-AMP-SO_3H.

Table S2. The effect of temperature on dye adsorption of MB, FS, Er, and MV over HKUST and HKUST-AMP-SO_3H.

Temperature (K)	Dye removal (%)					
	HKUST			HKUST-AMP-SO_3H		
	MB	FS	Er	MB	MV	Er
298	64	81	46	97	85	72
308	50	61	32	72	69	61
323	23	45	17	54	48	43

Table S3. The effect of adsorbent dosage on dye adsorption of MB, FS, Er, and MV.

Dose (mg)	Dye removal (%)					
	HKUST			HKUST-AMP-SO_3H		
	MB	FS	Er	MB	MV	Er
3	19	43	20	41	61	39
5	39	66	32	68	76	45
10	64	81	46	97	85	72
15	64	82	48	97	88	74
20	65	84	49	99	89	77

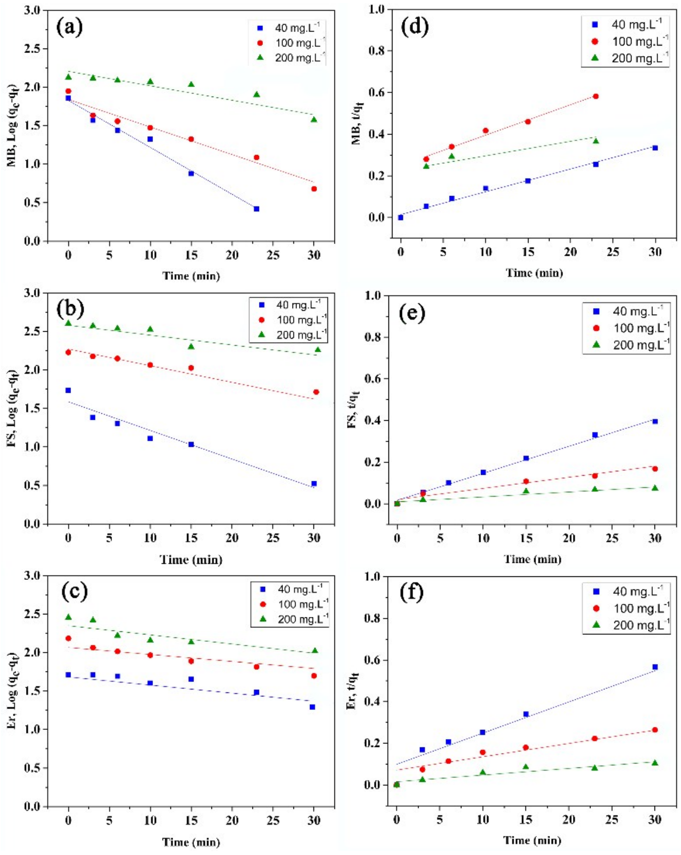

Fig. S6. Plots of pseudo-first-order kinetics over HKUST for a) MB, b) FS, and c) Er. Plots of pseudo-second-order kinetics over HKUST-AMP-SO_3H for d) MB, e) FS, and f) Er.

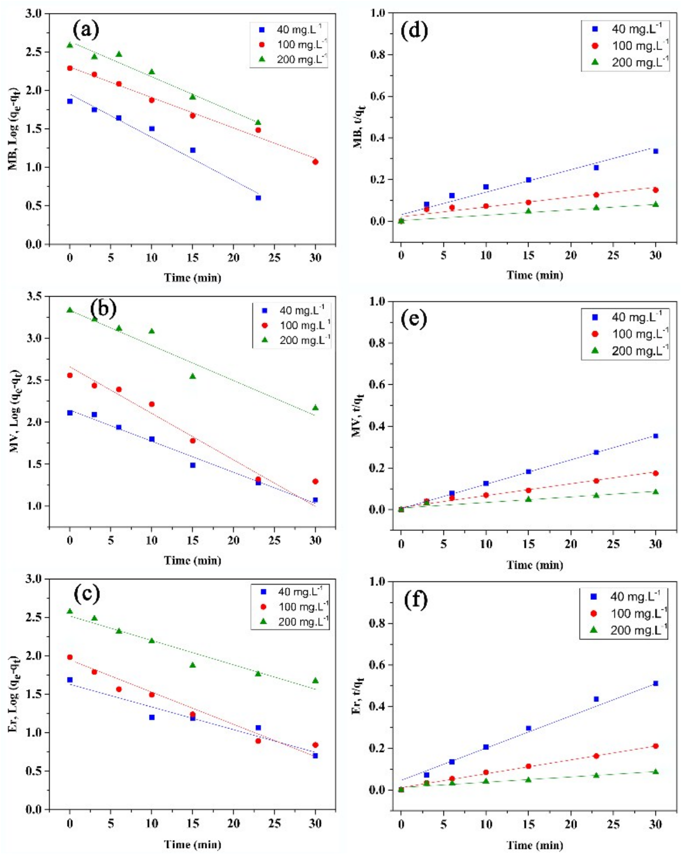

Fig. S7. Plots of pseudo-first-order kinetics over HKUST-AMP-SO_3H for a) MB, b) MV, and c) Er. Plots of pseudo-second-order kinetics over HKUST-AMP-SO_3H for d) MB, e) MV, and f) Er.

Table S4. Adsorption kinetics parameters of MB, FS, and Er using HKUST as adsorbent.

Dye	C_0(mg L^{-1})	pseudo-first-order			pseudo-second-order		
		K_1(min^{-1})	q_e(mg g^{-1})	R_2	K_2(g mg^{-1} min^{-1})	qe(mg g^{-1})	R_2
MB	40	0.140	67.28	0.9849	0.021	91.74	0.9994
	100	0.070	54.18	0.9357	0.011	53.76	0.9893
	200	0.012	128.20	0.973	0.001	142.85	0.9975
FS	40	0.085	38.69	0.9219	2.1×10^{-3}	59.17	0.9996
	100	0.027	125.89	0.849	1×10^{-3}	188.67	0.9911
	200	0.029	380.18	0.9791	0.6×10^{-3}	416.66	0.9930
Er	40	0.024	47.86	0.8626	0.002	66.66	0.9885
	100	0.020	114.81	0.9704	0.5×10^{-3}	156.25	0.9816
	200	0.027	218.77	0.9233	0.6×10^{-3}	312.5	0.990

Table S5. Adsorption kinetics parameters of MB, MV, and Er using HKUST-AMP-SO_3H as adsorbent.

Dye	C_0(mg L^{-1})	pseudo-first-order			pseudo-second-order		
		K_1(min^{-1})	q_e(mg g^{-1})	R_2	K_2(g mg^{-1} min^{-1})	qe(mg g^{-1})	R_2
MB	40	0.129	89.61	0.9566	3×10^{-3}	92.59	0.9973
	100	0.091	201.37	0.9874	1×10^{-3}	212.76	0.9942
	200	0.105	425.99	0.988	0.1×10^{-3}	400	0.9993
MV	40	0.002	129.12	0.9434	2×10^{-3}	204.08	0.9999
	100	0.003	153.12	0.9451	3×10^{-3}	175.43	0.9987
	200	0.1275	398.10	0.969	0.3×10^{-3}	370.37	0.9956
Er	40	0.025	24.54	0.9261	0.005	64.51	0.9976
	100	0.096	25.02	0.9235	0.006	149.25	0.9996
	200	0.073	323.59	0.938	0.5×10^{-3}	400	0.9956

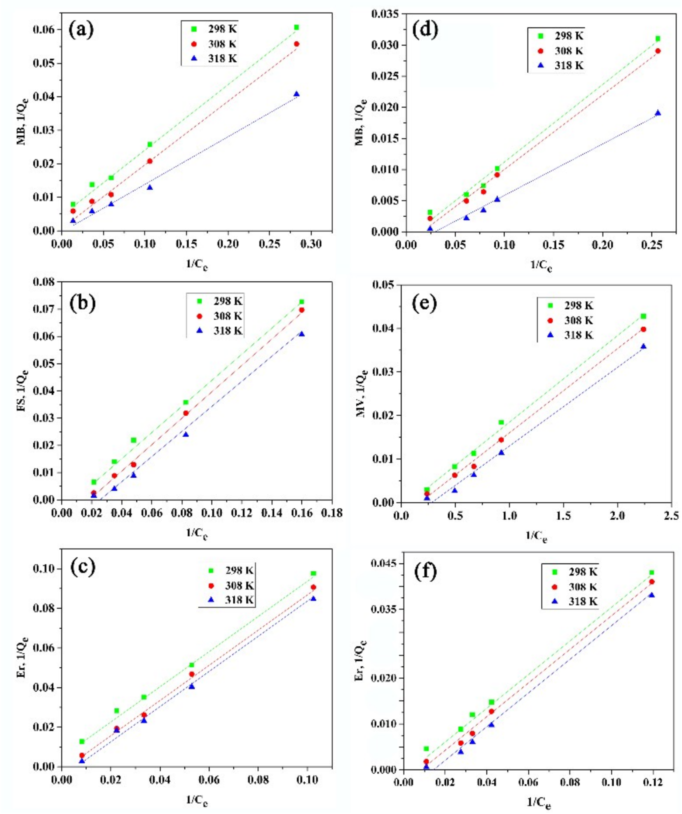

Fig. S8. Langmuir isotherm of adsorption over HKUST for a) MB, b) FS, and c) Er. Langmuir isotherm of adsorption over HKUST-AMP-SO_3H for d) MB, e) MV, and f) Er at 298, 308,318 ^{0}K.

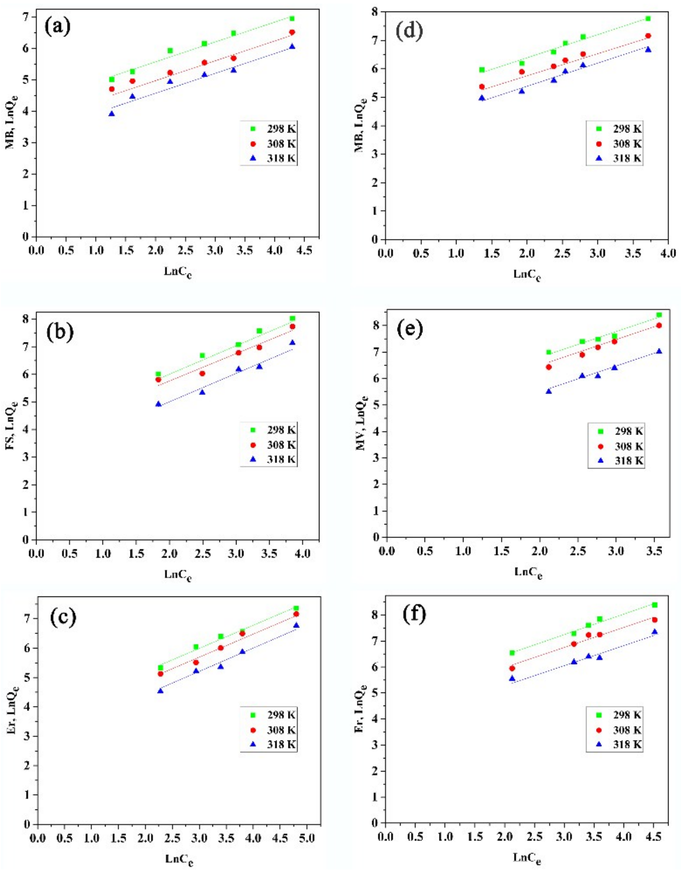

Fig. S9. Freundlich isotherm of adsorption over HKUST for a) MB, b) FS, and c) Er. Freundlich isotherm of adsorption over HKUST-AMP-SO_3H for d) MB, e) MV, and f) Er at 298, 308,318 ^{0}K.

Table S6. Isotherm parameters of MB, FS, and Er over HKUST.

Dye	T(K)	Langmuir				Freundlich		
		Q(mg/g)	K_L(L mg^{-1})	R_L	R_2	n_F	K_F	R_2
MB	298	238.09	0.0214	0.423	0.980	1.620	81.10	0.921
	308	224.65	0.0192	0.389	0.991	1.425	80.34	0.932
	318	204.21	0.0142	0.354	0.987	1.372	78.12	0.968
FS	298	243.90	0.0082	0.627	0.998	0.991	54.99	0.962
	308	234.02	0.0072	0.605	0.994	0.990	52.76	0.942
	318	210.54	0.0055	0.586	0.991	0.859	50.12	0.957
Er	298	200	0.0056	0.709	0.991	1.282	57.64	0.983
	308	183.21	0.0045	0.692	0.984	1.204	54.98	0.964
	318	176.09	0.0023	0.675	0.995	1.145	52.56	0.978

Table S7. Isotherm parameters of MB, MV, and Er over HKUST-AMP-SO_3H.

Dye	T(K)	Langmuir				Freundlich		
		Q(mg/g)	K_L(L mg^{-1})	R_L	R_2	n_F	K_F	R_2
MB	298	833.33	0.0096	0.598	0.993	1.240	119.02	0.974
	308	810.98	0.0084	0.431	0.986	1.178	110.56	0.962
	318	797.45	0.0071	0.367	0.991	1.023	105.98	0.979
MV	298	714.28	0.0706	0.201	0997	1.049	135.74	0.959
	308	706.23	0.0695	0.187	0.988	1.023	123.68	0.969
	318	695.87	0.0618	0.145	0.992	1.001	109.06	0.973
Er	298	833.33	0.0033	0.801	0.994	1.275	133.79	0.967
	308	813.98	0.0029	0.765	0.991	1.165	126.25	0.952
	318	798.23	0.0021	0.632	0.989	1.078	117.04	0.975

Table S8. Thermodynamic parameters of MB, FS, and Er adsorption over HKUST.

Dye	T(K)	LnK_0	ΔG°(kJ mol^{-1})	ΔH° (kJ mol^{-1})	ΔS° (J mol^{-1} K^{-1})
	298	1.43	-3.30		
MB	308	1.40	-2.91	-5.08	-5.09
	318	1.30	-2.73		
	298	1.04	-2.52		
FS	308	0.91	-2.14	-13.35	-36.04
	318	0.70	-1.74		
	298	0.65	-1.45		
Er	308	0.5	-1.19	-12.98	-38.10
	318	0.32	-0.81		

Table S9. Thermodynamic parameters of MB, MV, and Er adsorption over HKUST-AMP-SO_3H.

Dye	T(K)	LnK_0	ΔG°(kJ mol^{-1})	ΔH° (kJ mol^{-1})	ΔS° (J mol^{-1} K^{-1})
	298	2.22	-5.40		
MB	308	1.87	-4.73	-22.56	-57.58
	318	1.80	-4.14		
	298	6.12	-4.29		
MV	308	6.01	-4.13	-10.61	-15.35
	318	5.85	-3.96		
	298	1.01	-2.42		
Er	308	0.89	-2.21	-10.23	-25.89
	318	0.75	-1.89		

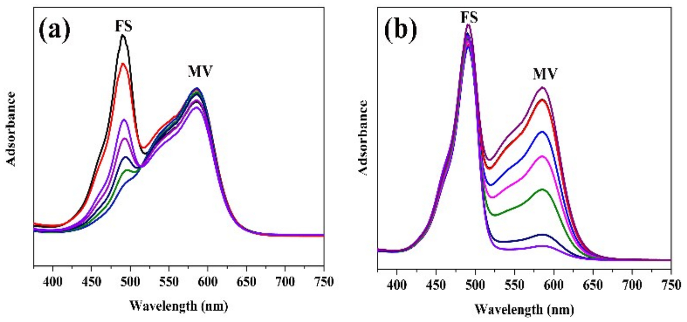

Fig. S10. The reverse performance of (a) HKUST and (b) HKUST-AMP-SO_3H for FS-MV mixture.

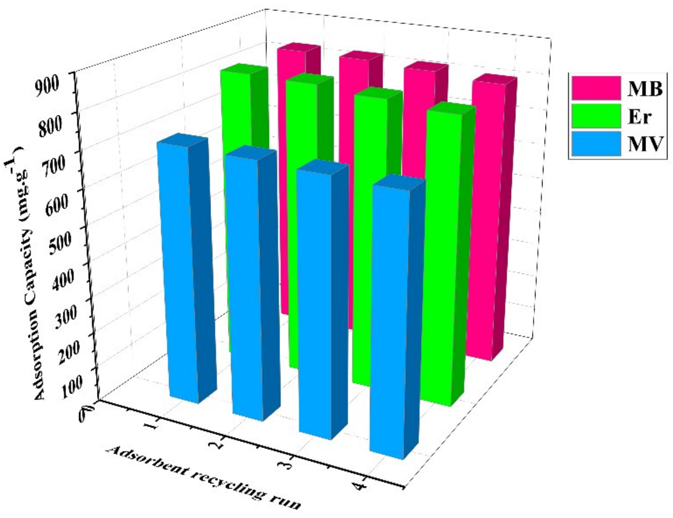

Fig. S11. The effect of recycling run on adsorption of MB, Er, and MV over HKUST-AMP-SO_3H.

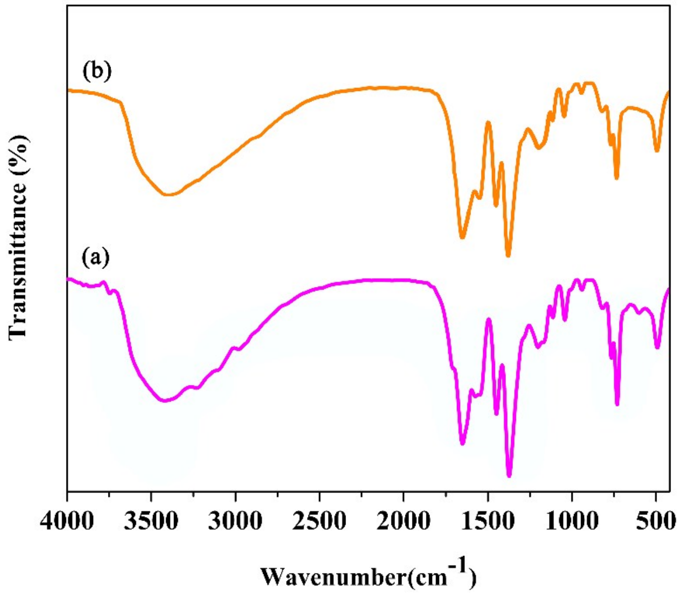

Fig. S12. FT-IR spectra of HKUST-AMP-SO_3H (a) before and (b) after using as an adsorbent.

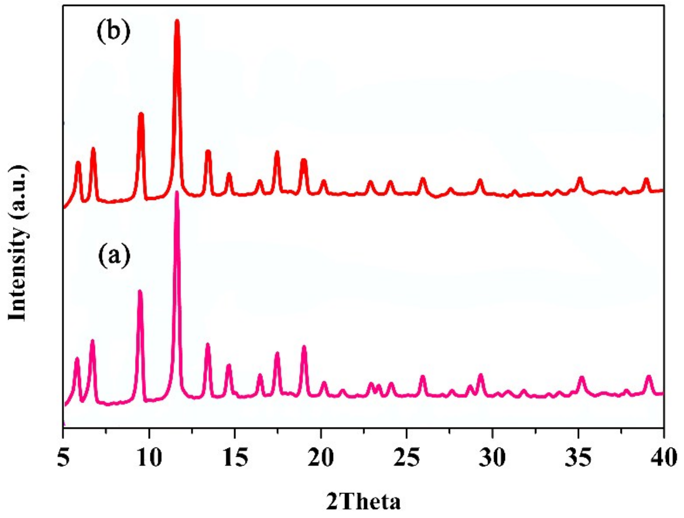

Fig. S13. XRD patterns of HKUST-AMP-SO_3H (a) before and (b) after using as an adsorbent.

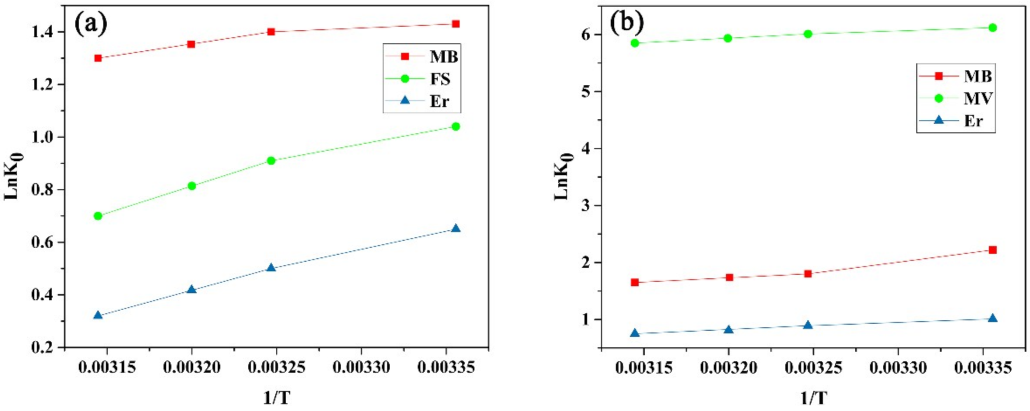

Fig. S14. Van't Hoff plots of used dye molecule over a) HKUST b) HKUST-AMP-SO_3H.
